# Editorial: Environmental Sustainability in Sports, Physical Activity and Education, and Outdoor Life

**DOI:** 10.3389/fspor.2022.853599

**Published:** 2022-02-21

**Authors:** Hans Kristian Hognestad, Richard Giulianotti, Holly Thorpe, Tommy Langseth, Bieke Gils

**Affiliations:** ^1^Department of Outdoor-Life Studies, Sport and Physical Education, University of South-Eastern Norway, Bø, Norway; ^2^School of Sport, Exercise and Health Sciences, Loughborough University, Loughborough, United Kingdom; ^3^School of Health, University of Waikato, Hamilton, New Zealand

**Keywords:** sport, environmental sustainability, outdoor activities in the natural environment, overheating, globalization

Environmental sustainability is one of the most urgent and complex “big issues” facing the contemporary world, as highlighted by its centrality to many of the United Nations' Sustainable Development Goals (SDGs). Environmental sustainability issues take many forms, including: climate change and carbon emissions, waste management, pollution and environmental degradation, the use of scarce natural resources such as water, the use of “dirty” and “clean” energy, the production and recycling of consumer items, and the impacts of modern living on biodiversity and the natural world.

Environmental sustainability is of critical importance to the fields of sports, physical activity and education, and outdoor life, in a whole range of ways. For example, the development of facilities and equipment for these activities requires the use of scarce natural resources, and has significant impacts on the natural environment; the staging of sport events often carries a large carbon footprint and generates vast material waste; physical exercise in many settings carries substantial environmental risks, such as air pollution or excess heat; and, participation in outdoor life activities, such as mountaineering or canoeing, may threaten local biodiversity and micro-climates. Issues of environmental sustainability thus have crucial significance for sport, physical activity and education, and outdoor living, at many different levels: in terms of geographical scale (local, national, regional, and global), in terms of types of activity involvement (as participants and players, spectators, organizers, and investors), and in terms of varieties of expertise and commitment (as professionals, elite competitors, amateurs, and informal participants).

The goal of this Research Topic is to advance knowledge and understanding of the environment and environmental sustainability in the three fields of sport, physical activity and education, and outdoor life. Our aim in developing this Research Topic has been to publish papers that draw on a diversity of disciplinary groundings, and to encompass a broad range of approaches relating to theory, methods, empirical content, and relevance for policy and practice.

The Research Topic builds on the conference on “Sport and the Environment” that was staged in 2019 under the auspices of the European Association of Sport Sociology, and which was hosted by the University of South-Eastern Norway (USN) at its campus in Bø, Telemark. Four of the Research Topic editors have positions at USN and were involved in the event's organization. The conference might be considered as a “breakthrough event”, in being the first to be staged by a leading international social science and sport association with a central thematic focus on the environment.

## The Research Topic Articles

The 11 papers in this collection share a focus on sport and the environment, yet topics vary from more theoretical analyses of the values of modern sport in a sustainability perspective via empirical considerations of material waste and carbon footprint to how sports organizations deal with higher demands for tackling environmental sustainability challenges.

Some international sport organizations seek to deal with environmental sustainability issues by adhering to international standards. Yet, as the article by Naess highlights, this approach has negative as well as positive sides for the sport, as illustrated through the case study of Formula E's pursuit of standardization certification. Naess likens the certification system to a “bazaar economy”, where there are various degrees of bargaining and clientelism in play. A very useful four-fold ideal-typical model is advanced, to register different types of environmental sustainability work relating to certification, and which can be applied across different sport organizations.

The “sport for development and peace” (SDP) sector, which uses sport for wider social change, has mushroomed across the world since the early 2000s. However, as the article by Giulianotti highlights, organizational stakeholders and academics with interests in SDP have been slow to examine how this global field can engage with environmental issues. In response, Giulianotti argues that a “socio-ecological” approach should be central to SDP activity and research, to recognize how environmental sustainability may only be tackled effectively if wider issues of social inequality and social justice are adequately addressed. This socio-ecological approach includes challenging “bourgeois environmentalism”, advancing democratization (such as through “associative democracy”), and transforming SDP into a “recognition space” that enables the full participation of the most marginal community members.

Indigenous peoples (also known by other terms, such as “first” or “native” peoples) are ethnic minorities which have been affected by the subsequent colonization of their lands by larger ethnic groups. The article by Skogvang examines the indigenous Sámi people in northern Norway, and the roles of festivals, as well as physical and cultural activities, in maintaining and reproducing their culturally distinctive forms of environmental awareness. Skogvang highlights in particular how Sámi festivals are intended to socialize and to educate young people with respect to indigenous cultural beliefs, customs, and practices, including in relation to the environment. Festivals have the further benefit of spreading awareness of the Sámi peoples, and their relationships to the environment, to wider audiences.

A topic rarely considered in relation to sport and the environment is the role of uniforms and clothing. In their article, Brice and Thorpe draw upon a feminist new materialist theoretical approach to consider the environmental impact of fitness clothing, particularly the highly popular style of activewear. Bringing interviews with women and their own lived experiences into conversation with Karen Barad's concept of entanglement, they examine human-clothing-environment relationships, revealing athleisure clothing itself is “an active, vital force that intra-acts with other non-human (and human) matter within the environment”. Focusing on both laundering and disposal practices, they demonstrate the ways in which feminist new materialisms encourage moves toward non-anthropocentric understandings of the sport-environment relationship, as well as new ethical practices in our everyday fitness lifestyles.

Sport-related tourism practices are another important topic related to sport travel, consumption and the environment. The paper by Rosenberg et al. applies micro-sociological perspective using Goffman's dramaturgical metaphors and concepts, while engaging empirical data from an ethnographic study of five different multi-day trips in Norway (using skiing, hiking, or biking as modes of travel), to reveal how the “different actors understood, operationalized and practiced elements of sustainability in their everyday lives while on the trips”. This examination of nature-based adventure tourism highlights the fascinating interplay between tourists, guides, adventure activities and nature, and ultimately reveals performances of “light” sustainability. The work being done by those in nature-based sport economies, and the complexities and contradictions they must navigate, is an important topic deserving further investigation.

An important and often overlooked aspect in studies on sport and sustainability, according to Braksiek et al. are sport participants' attitudes toward environmentally friendly behavior. While more attention has been paid to such behaviors in research on elite sport contexts, the authors identify a lack of such research at the sport participation grassroots level. Using Icek Ajzen's Theory of Planned Behavior (1991) from the field of social psychology, the researchers surveyed 3,036 male and female members in German community sport clubs for three antecedents of behavior; participants' attitudes toward the behavior, subjective norms, and behavioral intentions. While differences between men and women were detected in the antecedents-intention relationship, the study found that the three antecedents successfully predicted the behavioral intentions of sports club members to act environmentally friendly, concluding that the Theory of Planned Behavior can be fully applied to sports club members and to the grassroots sports context.

“Can sport be sustainable?” is a question rarely asked directly in research on sport and sustainability, though a crucial starting point for research and discussions on sport and sustainability. “It depends” answers Tangen, in an elaborate and personal essay in which he weaves together a colorful tapestry of literatures, illustrative examples and reflections while building on Niklas Luhmann's system theory. According to Tangen, the modern sport ethos with its emphasis on breaking records, and thus “unlimited growth”, embodies, reflects and espouses modernity's illnesses at the heart of the current climate crisis. Can the hypertrophic growth of sport and its associated climate and social injustices be counteracted? Not unless, Tangen writes, modern society scales down and moves toward a more “simple but global, low-tech based society where playful, physical contests may take place locally and be sustainable” (p.14).

Eriksen has written extensively on the concept of overheating in order to understand accelerated change as a major effect of global capitalism. Sport may be seen as both a metaphor and a major driver for this development. In his contribution to this issue he argues that the upward spiral of improved achievement in modern sport can be seen both as an effect of the global market and a carrier of modern values of development and growth. By drawing on the concept of “the red queen effect”, referring to forms of competition among species in which improvement is necessary in order to survive, Eriksen scrutinizes the paradoxes inherent in modern sport and applies his analysis as a critique of an unsustainable economic growth.

While there has been an increasing effort to build environmentally friendly sport stadiums since the millenium, the emphasis has been on constructing new arenas as older stadiums are seen as less energy efficient, in line with a widespread belief in international architecture. Wergeland and Hognestad show in their contribution how this belief has served predominantly the commercial interests embedded in the stadiums, as “new” sells more easily than “old”. They provide a critical discussion of these tendencies, which have implied a principle of growth both in revenue and in size. By dwelling on two historical stadiums which have been threatened with demolition in recent decades—Stadio Flamino in Rome and Tynecastle Park in Edinburgh—Wergeland and Hognestad highlight the social and environmental importance of reusing stadiums for a greener future.

While research on the sustainability and environmental impact of sport mega events has been quite extensive, smaller and more local events have not been studied to the same degree. Jensen analyses the conceptualization of sustainability regarding sport events in 22 different municipalities in Norway. He finds that sustainability is an “unfinished puzzle” for local policy makers and that there are great variations between the municipalities with regard to how they conceptualize sustainability in their masterplans and how this is translated (or not translated) into event policies. Jensen's analysis reveals the complexity local policymakers face when there is demand for both more events and increased sustainability measures.

Many lifestyle sports participants see themselves as “green”. In their article, Langseth and Wyff study surfers' environmental attitudes and action. While they found that most surfers have environmental attitudes, they also found a gap between their expressed attitudes and their actions. This is especially evident in connection with extensive long distance travels, when the search for the most interesting spots, waves and locations tend to overrule environmental concerns associated with such travels. Langseth and Wyff argue that this discrepancy can be seen as the result of a cultural dissonance, as “being green” and extensive travels both have value and are recognized as key to participation within surf cultures.

## Author Contributions

All authors listed have made a substantial, direct, and intellectual contribution to the work and approved it for publication.

## Conflict of Interest

The authors declare that the research was conducted in the absence of any commercial or financial relationships that could be construed as a potential conflict of interest.

## Publisher's Note

All claims expressed in this article are solely those of the authors and do not necessarily represent those of their affiliated organizations, or those of the publisher, the editors and the reviewers. Any product that may be evaluated in this article, or claim that may be made by its manufacturer, is not guaranteed or endorsed by the publisher.

